# Childhood leukaemia in North West England 1954-1977: epidemiology, incidence and survival.

**DOI:** 10.1038/bjc.1981.51

**Published:** 1981-03

**Authors:** J. M. Birch, R. Swindell, H. B. Marsden, P. H. Morris Jones

## Abstract

The annual incidence of leukaemia among children aged up to 14 years as estimated by the Manchester Children's Tumour Registry has been analysed for the 24 years 1954-1977. A significant increase in acute lymphoid leukaemia (ALL) was found, while the incidence of acute myeloid leukaemia (AML) remained constant. Other types of leukaemia were too rare to be analysed separately. The increase in ALL was concentrated among boys in the 1--5-year age group. Analysis with respect to initial white-cell count showed the increase to be more pronounced in children with initial white cell counts of 1-5 x 10(4)/microliters. The proportion of cases presenting in Lancashire compared with Greater Manchester did not change during the study period. The distribution of cases with respect to social class and socio-economic group of the parents also remained constant. Due to advances in the treatment of childhood ALL survival improved considerably during the study period and no increase in mortality was seen.


Br. J. Cancer (1981), **43**, 324

### CHILDHOOD LEUKAEMIA IN NORTH WEST ENGLAND 1954–1977: EPIDEMIOLOGY, INCIDENCE AND SURVIVAL

J. M. BIRCH*, R. SWINDELL†, H. B. MARSDEN* AND P. H. MORRIS JONES‡

*From the *Children's Tumour Registry, Department of Epidemiology and Social Research, †Department of Medical Statistics, Christie Hospital and Holt Radium Institute, University of Manchester, and ‡Royal Manchester Children's Hospital, Department of Child Health (Paediatric Oncology), University of Manchester*

Received 23 September 1980 Accepted 21 November 1980

**Summary.—The annual incidence of leukaemia among children aged up to 14 years as estimated by the Manchester Children's Tumour Registry has been analysed for the 24 years 1954–1977. A significant increase in acute lymphoid leukaemia (ALL) was found, while the incidence of acute myeloid leukaemia (AML) remained constant. Other types of leukaemia were too rare to be analysed separately. The increase in ALL was concentrated among boys in the 1–5-year age group. Analysis with respect to initial white-cell count showed the increase to be more pronounced in children with initial white cell counts of $1–5 \times 10^4/\mu l$. The proportion of cases presenting in Lancashire compared with Greater Manchester did not change during the study period. The distribution of cases with respect to social class and socio-economic group of the parents also remained constant. Due to advances in the treatment of childhood ALL survival improved considerably during the study period and no increase in mortality was seen.**

MALIGNANT DISEASE is the second commonest cause of death in children aged 1–14 years, and is exceeded only by accidents (Office of Population Censuses and Surveys, 1978). About one-third of all childhood malignancies are leukaemias. Although rare, leukaemia is nevertheless an important paediatric problem. Population data which combine diagnostic accuracy with a high level of ascertainment are uncommon, but the Manchester Children's Tumour Registry (MCTR) has these qualities, and represents a unique time series for detailed study (Young & Miller, 1975). A rise in the incidence of childhood acute lymphoid leukaemia (ALL) in North West England has previously been reported (Birch *et al.*, 1979). The features of this increase have now been analysed, together with trends in survival, and the results of this work are reported below.

### MATERIALS AND METHODS

The MCTR collects clinical and pathological details of all cases of malignant disease occurring in children aged 0–14 years who are resident in the North Western Regional Health Authority (NWRHA) area of England (Manchester Regional Health Board (MRHB) area before 1974) and is described in detail elsewhere (Birch *et al.*, 1980; Leck *et al.*, 1976). The records, including diagnoses, of all cases of leukaemia registered during the 24 years 1954–1977 have been reviewed for the current study, taking all the available clinical and pathological information into account. The majority of marrow specimens were examined by haematologists at the Royal Manchester Children's Hospital, which serves as the regional centre for the treatment of childhood leukaemia.

It is now clear that most if not all childhood leukaemias historically classified as "stem-cell leukaemia" are lymphatic in origin and with modern specific staining

Address for reprints: Dr Jillian M. Birch, Department of Epidemiology and Social Research, Children's Tumour Registry, Christie Hospital, Manchester M20 9BX.

techniques currently in use this diagnosis is rarely made (Hayhoe *et al.*, 1964; Shaw, 1976; Fernbach, 1977). Early cases included in the present series, which were originally diagnosed as "stem-cell leukaemia", have been reclassified as ALL, providing this was consistent with other clinical features.

The annual incidence of ALL and acute myeloid leukaemia (AML) was calculated using estimates of the mid-year populations of children aged under 15 years resident in the NWRHA (MRHB before 1974) as the denominators, and trends in incidence examined by linear regression. [A cusum plot of the ALL data has previously been shown (Birch *et al.*, 1979).] Annual incidence of ALL among males and females and for the age groups $0<1$ year, $1<5$ years, $5<10$ years and $10<15$ years was calculated, using relevant population estimates as denominators, and incidence trends for these groups examined, using a cumulative sum (cusum) technique with a target mean calculated from the first 10 years' incidence (Woodward & Goldsmith, 1964; Chaput de Saintonge & Vere, 1974). The effect of a cusum plot is to magnify a change in trend in order to facilitate its detectability by a simple graphical method; the actual change in incidence is less dramatic than would appear from the figures. The cusum test used had a V-mask scheme of $\theta = 26{\cdot}5°$ and a lead distance of 1 year. The vertical scales in Figs 2, 3 and 4 are related to the standard deviation of the target mean, and are chosen so that the cusum test can be directly applied.

ALL can be divided into prognostic groups according to initial white-cell count (WCC) at presentation. Low initial WCC is associated with a good prognosis and high with a poor prognosis. Cases of ALL included were therefore divided into 3 groups with respect to WCC, and incidence trends among these groups examined as described above. The WCC groups were as follows: $<10^4/\mu l$; $1\text{–}5\times10^4/\mu l$; $>5\times10^4/\mu l$. There were too few cases of other types of leukaemia for separate analysis.

The occupation of the father of each child with ALL was obtained from their birth certificates. Social class and socio-economic groups were allocated according to the Registrar General's classification (1970) and the distribution of these for the 1st 12-year period compared with the 2nd 12-year period.

The proportion of cases of ALL presenting annually from Lancashire Area Health Authority (excluding Ormskirk) was compared with that from Greater Manchester. Cheshire and the North Lake District could not be included in this analysis, as parts of these areas were lost from the region after reorganization in 1974.

Kaplan–Meier survival curves (Kaplan & Meier, 1958) were calculated for children with ALL presenting in 4 6-year periods: 1954–1959; 1960–1965; 1966–1971 and 1972–1977, and compared by the log-rank test (Peto *et al.*, 1977). Similar curves were calculated for AML.

### RESULTS

Total numbers of each leukaemic cell type which presented during the study period are reported elsewhere (Birch *et al.*, 1980). The Table shows numbers of cases of males and females in various age groups for 4 6-year periods. The population increased progressively during the first 3 of these periods and then decreased during the fourth period to equal that of the first.

Linear-regression analysis showed no

TABLE.—*Numbers of cases of acute lymphoid leukaemia by age-group, sex and calendar period*

| | Age (years) | | | | | | | |
|---|---|---|---|---|---|---|---|---|
| | Males | | | | Females | | | |
| Year of presentation | 0–1 | 1–5 | 5–10 | 10–15 | 0–1 | 1–5 | 5–10 | 10–15 |
| 1954–1959 | 3 | 36 | 26 | 14 | 1 | 36 | 12 | 9 |
| 1960–1965 | 6 | 46 | 29 | 10 | 2 | 27 | 23 | 10 |
| 1966–1971 | 3 | 51 | 26 | 20 | 3 | 40 | 18 | 10 |
| 1972–1977 | 0 | 57 | 33 | 19 | 3 | 39 | 16 | 10 |

FIG. 1.—Annual incidence of ALL per $10^6$ person years. Regression line with 95% confidence interval is shown.

FIG. 2.—Cusum plots of annual incidence of ALL per $10^6$ person years among boys and girls. Target means (boys, $27{\cdot}6 \pm 5{\cdot}3$; girls, $20{\cdot}5 \pm 6{\cdot}6$) calculated from first 10 years' incidence.

change in the incidence of AML with time, but a significant ($P < 0{\cdot}02$) increase in the annual incidence of ALL, from about 23 per million person years to about 29 per million person years (Fig. 1) was found. There were only 13 cases of chronic myeloid leukaemia: no significant trend could be established, but it is of interest that 9 of these cases presented after 1970.

Cusum plots of males (379 cases) and females (259 cases) with ALL (Fig. 2) show the increase to be considerably greater among males. Fig. 3 shows the various age groups separately. There were only 21 cases aged under 1 year. Although this is too few to show a clear trend, there was a decrease rather than an increase in incidence in this age group. The most marked increase was seen in the 1–5-year-olds (332 cases). No significant trends with time were seen for the 5 < 10-year group (183 cases) nor the 10 < 15-year group (102 cases). Among the WCC groups (Fig. 4) a significant increase was seen in the $1\text{–}5 \times 10^4$ group (189 cases). In the $< 10^4$ group (313 cases) an increase was seen between 1970 and 1973, which then tailed off, and the overall effect was not significant. No trend with time was seen in the $> 5 \times 10^4$ group (133 cases).

Analysing the sexes separately, the increase was significant in males aged 1–5 years, and in males with a WCC of $1\text{–}5 \times 10^4$. The respective groups of females did not show a significant increase, although these groups were small.

The ratio of cases presenting annually from Lancashire to those from Greater Manchester remained constant throughout the study period. The percentages were ~32% and 68% respectively and reflected the relative proportion of the entire child population.

Distribution of social class and socio-economic group in 1954–1965 was the same as for the period 1966–1977. Social-class distribution was as follows: 1 and 2, 16%; 3, 55%; 4 and 5, 24%; and 5% not known. The distribution did not differ from the population as a whole.

Survival among children with ALL improved considerably during the 24 years under study (Fig. 5). Five-year survival

FIG. 3.—Cusum plots of annual incidence of ALL per $10^6$ person years among various age groups. Target means (0–1, $15{\cdot}2 \pm 14{\cdot}3$; 1–5, $42{\cdot}7 \pm 12{\cdot}9$; 5–10, $21{\cdot}5 \pm 9{\cdot}6$; 10–15, $11{\cdot}8 \pm 6{\cdot}1$) calculated from first 10 years' incidence.

FIG. 4.—Cusum plots of annual incidence of ALL per $10^6$ person years among various WCC groups. Target means $< 10^4$, $12{\cdot}0 \pm 3{\cdot}1$; $1\text{–}5 \times 10^4$, $6{\cdot}7 \pm 2{\cdot}3$; $> 5 \times 10^4$, $5{\cdot}2 \pm 2{\cdot}6$) calculated from first 10 years' incidence.

increased from 1·5% in 1954–1959 to 32·6% in 1972–1977. The trend was highly significant ($P < 0{\cdot}0001$). Seven patients from the first 12 years are still alive and well, though during this time treatment was minimal and the disease usually fatal within 2–3 years. Similar improvements in survival have not been seen among AML cases, and only 2 patients out of 121 survived 5 years. Three-year survival improved from 3·2% to 16·1% ($P < 0{\cdot}005$).

There were 16 children with Down's syndrome in the present series, all of whom had ALL. One case of Down's would have been expected, which gives an

FIG. 5.—Survival of children with ALL in 4 consecutive 6-year periods ($P < 0{\cdot}0001$).

excess risk in accord with other estimates (Miller, 1970).

### DISCUSSION

A significant increase in the incidence of ALL in children resident in North West England has been observed. It is unlikely that this increase is due to improved ascertainment, for reasons discussed below, and we believe the increase to be real.

The MCTR has maintained a high level of ascertainment throughout its existence (Leck *et al.*, 1976) and accuracy of diagnosis has always been one of the Registry's main concerns. As stated above, cases which were previously classified as "acute stem-cell" leukaemia were added to the ALL for the purpose of this study. It is possible that a small number of these cases could have been myeloid in origin, and this could lead to errors in estimating incidence. However, as only 4 cases of "stem-cell" leukaemia have been registered since 1970, such errors would tend to exaggerate the incidence of ALL in the early part of the study period and hence the increase would appear *less* marked than it really is.

The observation that the increase is concentrated in males, in one particular age group and one particular WCC group argues against the increase being the result of changes in ascertainment. There is no apparent reason why boys should be ascertained rather than girls, nor why ascertainment should have improved among 1–5-year-olds and among children with WCCs in one range rather than another. If it is accepted that this increase is real, an explanation must be sought. The population of genetically susceptible children may have increased, environmental leukaemogens may be more prevalent, or a combination of these circumstances may exist.

The increase showed no striking geographical variations within the North West region, and a report by Stiller & Draper (1980) suggests that our observations are part of a national increase. If environmental leukaemogens are responsible for this rise in incidence, then these must be widely and evenly spread with respect to place and to social class. Our data show that the rise began about 1970 and the effect may be due to a widespread change in social habits or medical practice which took place in the mid-1960s. The increase is most marked in the 1–5-year age range, which encompasses the peak in incidence which characterizes ALL in white child populations. Perhaps the aetiology of leukaemia in this age group is different from that among older children, and prenatal influences may be more important.

Boys predominate in a number of reported series of leukaemia in childhood (Teppo *et al.*, 1975; Young & Miller, 1975; Ericsson *et al.*, 1978; Li *et al.*, 1980) and the greater incidence among boys in the present series has exaggerated this feature. Boys may be genetically more susceptible to the development of ALL, or perhaps the male foetus experiences a more hostile uterine enviornment than the female, with a resultant susceptibility to environmental leukaemogens, prenatally and postnatally.

Clinically and haematologically, ALL appears to be a group of related diseases (Thierfelder *et al.*, 1979; Kumar *et al.*, 1979). The present increase in ALL is more marked in children with an initial WCC of $1\text{–}5 \times 10^4/\mu l$. Epidemiologically the various sub-groups would appear to behave differently, and may be aetiologically distinct. With present haematological, histochemical and immunological techniques the sub-types may be defined more precisely, and it will be interesting to study these groups epidemiologically in the light of current biological knowledge.

Few cancer registries with reliable data have been established long enough to make studies of temporal trends in incidence. Studies of this kind for childhood cancer have been carried out in Sweden and Finland (Teppo *et al.*, 1975; Ericsson *et al.*, 1978). In neither of these series was an increase in leukaemia incidence seen, but the different cell types were not considered separately. However, the overall incidence of leukaemia in these two countries, and among U.S. whites (Young & Miller, 1975), is higher than in the present study, and it may be that the incidence in the North West region, and probably the United Kingdom as a whole, is rising to a level already established in these other populations.

During the period when the increase in incidence of ALL took place there was a considerable improvement in survival. The increase would not, therefore, be reflected in mortality data. This observation not only strikes a note of optimism in what might otherwise be an alarming situation, but emphasizes the need for efficient cancer registration. With current advances in therapy for a number of malignant diseases, the monitoring of cancer mortality is no longer valid as a means of detecting changes in incidence.

The great improvement in survival among ALL patients has been achieved as a result of the introduction of standardized protocols involving multi-agent chemotherapy and central-nervous-system prophylaxis. During this time also paediatric oncology developed as a specialty and treatment of cases was centralized.

Prospective studies are currently under way to investigate further the epidemiology of leukaemia in childhood.

The Manchester Children's Tumour Registry is supported by the Cancer Research Campaign.

### REFERENCES

BIRCH, J. M., MARSDEN, H. B. & SWINDELL, R. (1979) Incidence of acute leukaemia of childhood in North West England. *Lancet*, ii, 854.

BIRCH, J. M., MARSDEN, H. B. & SWINDELL, R. (1980) Incidence of malignant disease in childhood: A 24-year review of the Manchester Children's Tumour Registry data. *Br. J. Cancer*, **42**, 215.

CHAPUT DE SAINTONGE, D. M. & VERE, D. W. (1974) Why don't doctors use cusums? *Lancet*, i, 120.

ERICSSON, J. L-E., KARNSTRÖM, L. & MATTSSON, B. (1978) Childhood cancer in Sweden 1958–1974. I. Incidence and mortality. *Acta Paediatr. Scand.*, **67**, 425.

FERNBACH, D. J. (1977) Natural history of acute leukaemia. In *Clinical Pediatric Oncology*. Eds Sutow *et al.* St Louis: Mosby Co. p. 153.

HAYHOE, F. G. J., QUAGLINO, D. & DOLL, R. (1964) The cytology and cytochemistry of acute leukaemias: A study of 140 cases. *MRC special report series No. 304*. London: HMSO.

KAPLAN, E. L. & MEIER, P. (1958) Non-parametric estimation from uncomplete observations. *J. Am. Stat. Assoc.*, **58**, 457.

KUMAR, S., CARR, T. F., EVANS, D. I. K., MORRIS JONES, P. & HANN, I. M. (1979) Prognostic significance of cell surface markers in childhood acute lymphoblastic leukaemia. *Clin. Lab. Haematol.*, **1**, 121.

LECK, I., BIRCH, J. M., MARSDEN, H. B. & STEWARD, J. K. (1976) Methods of classifying and ascertaining children's tumours. *Br. J. Cancer*, **34**, 69.

LI, F. P., JIN, F., TU, C. T. & GAO, Y. T. (1980) Incidence of childhood leukaemia in Shanghai. *Int. J. Cancer*, **25**, 701.

MILLER, R. W. (1970) Neoplasia and Down's syndrome. *Ann. N.Y. Acad Sci.*, **171**, 637.

OFFICE OF POPULATION CENSUSES AND SURVEYS (1978) *Mortality Statistics: Childhood and Maternity.* Series DH3, **5**, London: HMSO.

PETO, R., PIKE, M. C., ARMITAGE, P. & 7 others (1977) Design and analysis of randomized clinical trials requiring prolonged observation of each patient: II Analysis and examples. *Br. J. Cancer*, **35**, 1.

SHAW, M. T. (1976) The cytochemistry of acute leukaemia: A diagnostic and prognostic evaluation. *Semin. Oncol.*, **3**, 219.

STILLER, C. A. & DRAPER, G. J. (1980) Variations in the incidence of childhood leukaemia. *J. Epidemiol. Commun. Hlth*, **34**, 152.

TEPPO, L., SALONEN, T. & HAKULINEN, T. (1975) Incidence of childhood cancer in Finland. *J. Natl Cancer Inst.*, **55**, 1065.

THIERFELDER, S., RODT, H., THIEL, E. and 5 others (1979) Immunologic markers for classification of leukaemias and non-Hodgkin's lymphomas. *Recent Results Cancer Res.*, **69**, 41.

WOODWARD, R. H. & GOLDSMITH, P. L. (1964) Mathematical and statistical techniques for industry. I.C.I. Ltd. Monograph No. 3. *Cumulative Sum Techniques*. Edinburgh: Oliver & Boyd.

YOUNG, J. L. & MILLER, R. W. (1975) Incidence of malignant tumours in U.S. children. *J. Pediatr.*, **86**, 254.


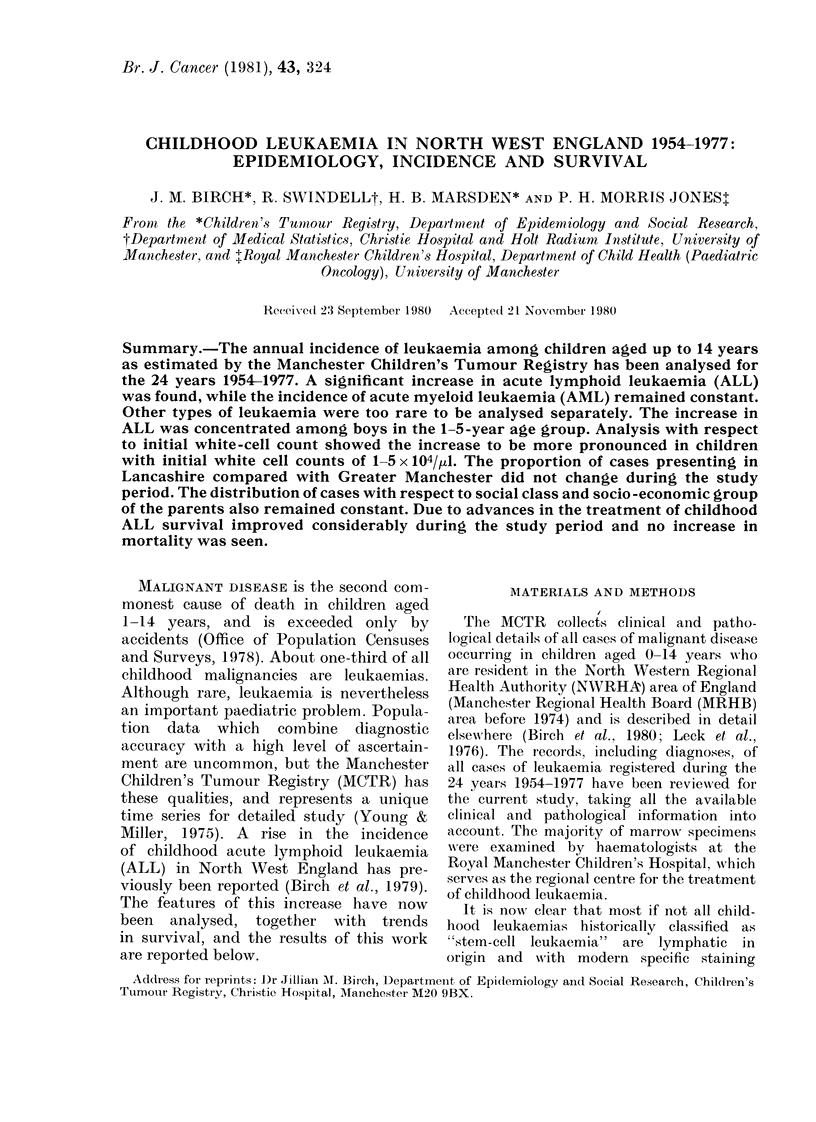

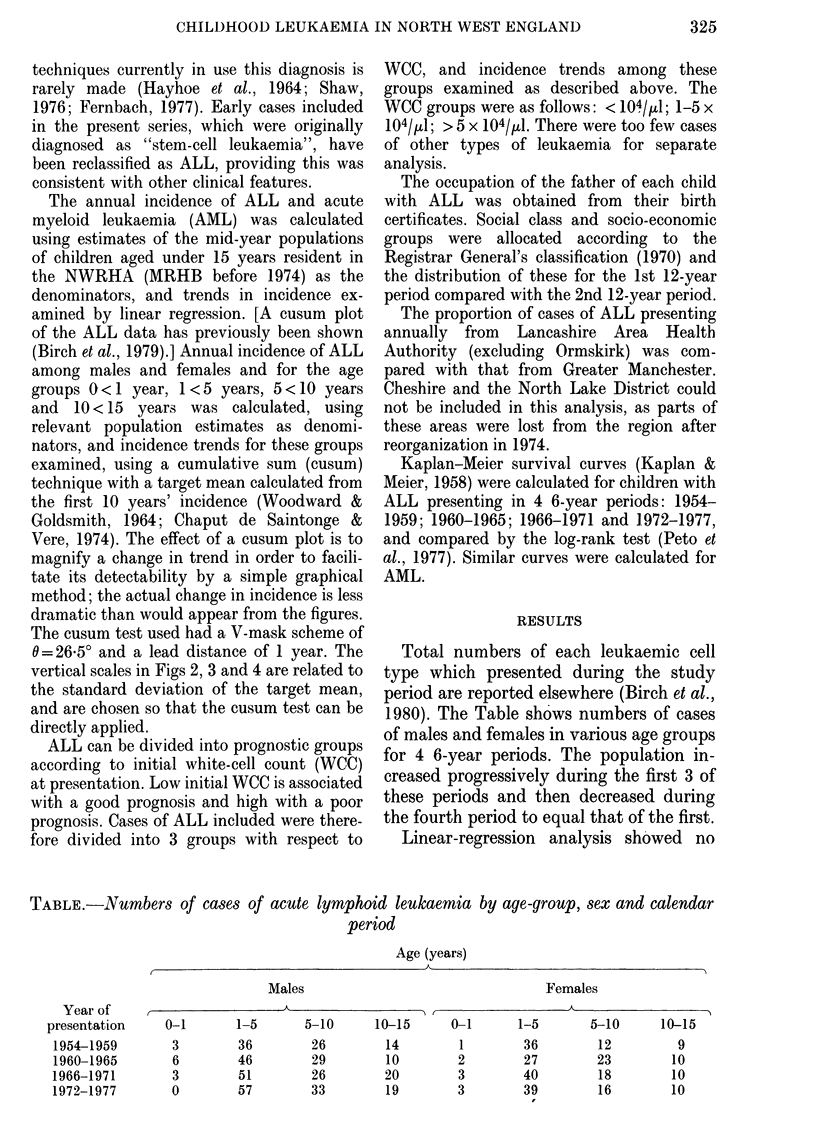

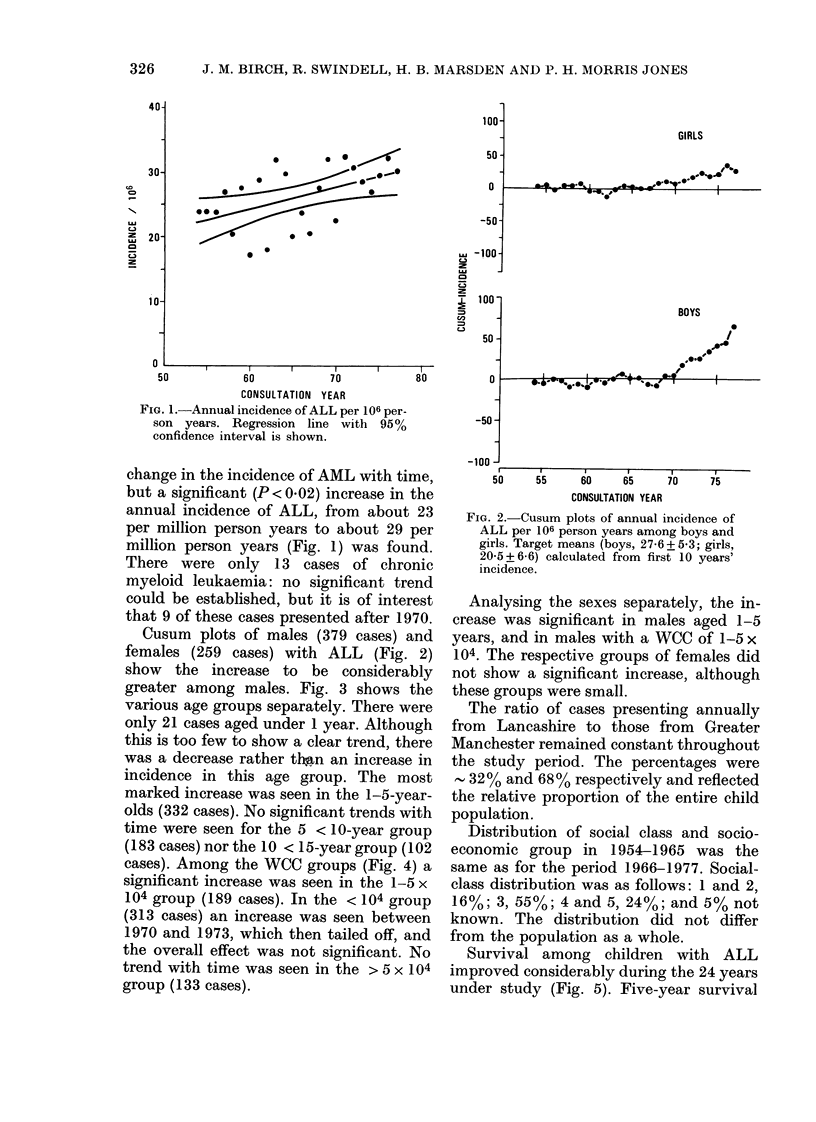

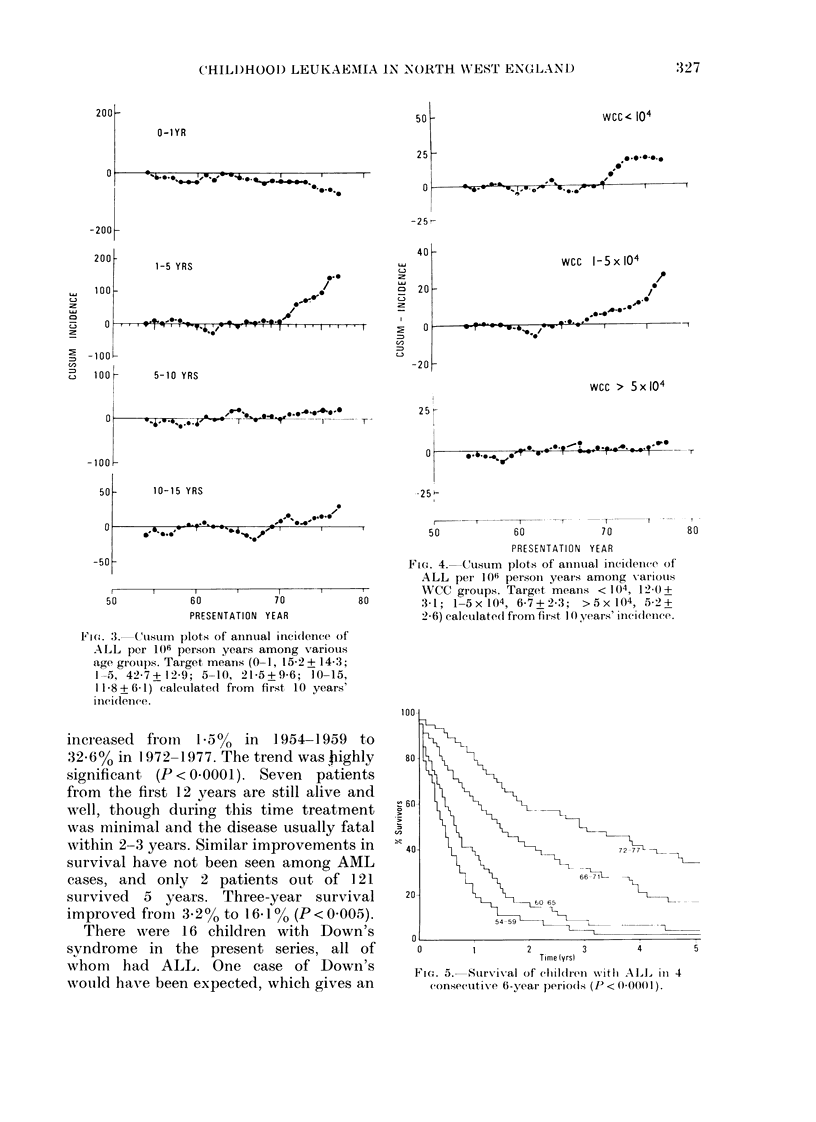

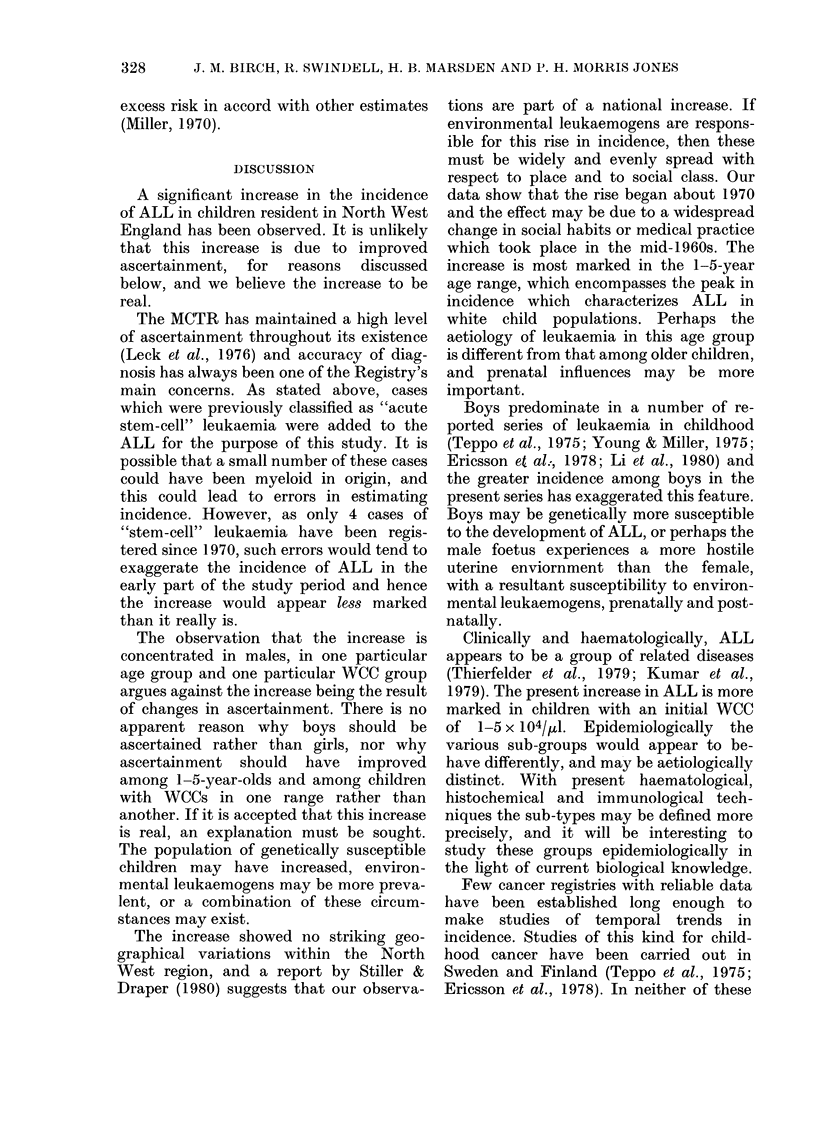

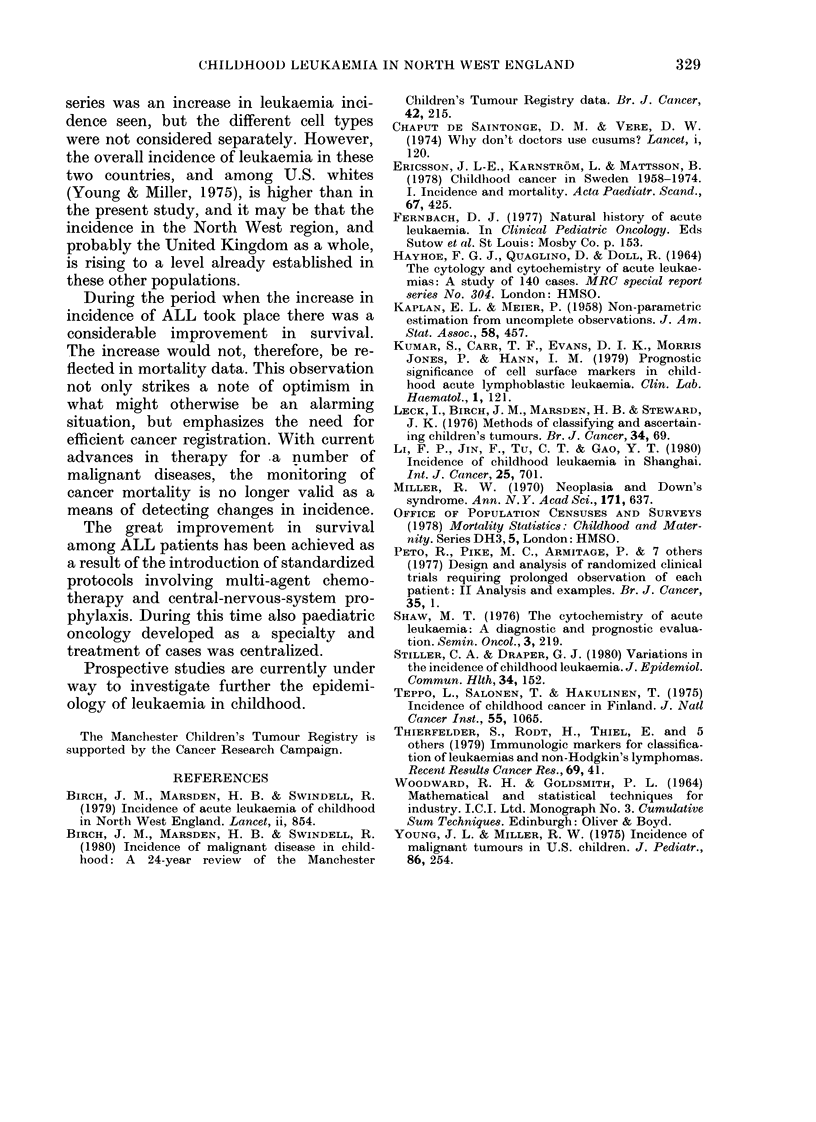


## References

[OCR_00688] Birch J. M., Marsden H. B., Swindell R. (1979). Acute lymphoid leukaemia of childhood in North-West England.. Lancet.

[OCR_00693] Birch J. M., Marsden H. B., Swindell R. (1980). Incidence of malignant disease in childhood: a 24-year review of the Manchester Children's Tumour Registry data.. Br J Cancer.

[OCR_00701] Chaput de Saintonge D. M., Vere D. W. (1974). Why don't doctors use cusums?. Lancet.

[OCR_00708] Ericsson J. L., Karnström L., Mattsson B. (1978). Childhood cancer in Sweden, 1958-1974. I. Incidence and mortality.. Acta Paediatr Scand.

[OCR_00730] Kumar S., Carr T. F., Evans D. I., Morris-Jones P., Hann I. M. (1979). Prognostic significance of cell surface markers in childhood acute lymphoblastic leukaemia.. Clin Lab Haematol.

[OCR_00735] Leck I., Birch J. M., Marsden H. B., Steward J. K. (1976). Methods of classifying and ascertaining children's tumours.. Br J Cancer.

[OCR_00740] Li F. P., Jin F., Tu C. T., Gao Y. T. (1980). Incidence of childhood leukemia in Shanghai.. Int J Cancer.

[OCR_00761] Shaw M. T. (1976). The cytochemistry of acute leukemia: a diagnostic and prognostic evaluation.. Semin Oncol.

[OCR_00771] Teppo L., Salonen T., Hakulinen T. (1975). Incidence of childhood cancer in Finland.. J Natl Cancer Inst.

[OCR_00788] Young J. L., Miller R. W. (1975). Incidence of malignant tumors in U. S. children.. J Pediatr.

